# Hypocretin-1 receptor antagonism improves inhibitory control during the Go/No-Go task in highly motivated, impulsive male mice

**DOI:** 10.1007/s00213-024-06628-3

**Published:** 2024-06-18

**Authors:** Jeremy Metha, Yijun Ji, Clemens Braun, Janet R. Nicholson, Luis De Lecea, Carsten Murawski, Daniel Hoyer, Laura H. Jacobson

**Affiliations:** 1 Florey Institute of Neuroscience and Mental Health, Parkville, VIC 3052 Australia; 2 Department of Finance, The University of Melbourne, Parkville, VIC 3052 Australia; 3 Department of Biochemistry and Pharmacology, School of Biomedical Sciences, Faculty of Medicine, Dentistry and Health Sciences, The University of Melbourne, Parkville, VIC 3052 Australia; 4 Drug Discovery Sciences, Boehringer Ingelheim Pharma GmbH & Co. KG, 88397 Biberach, Germany; 5 CNS Diseases Research, Boehringer Ingelheim Pharma GmbH & Co. KG, 88397 Biberach, Germany; 6 Department of Psychiatry and Behavioural Sciences, Stanford University, Stanford, CA 94305 USA; 7 Department of Molecular Medicine, The Scripps Research Institute, La Jolla, CA 92037 USA; 8 Circadian Misalignment and Shift Work Laboratory, Turner Institute for Brain and Mental Health, School of Psychological Sciences, Monash University, Notting Hill, VIC, 3162, Australia

**Keywords:** Hypocretin, Orexin, Impulsivity, Inhibitory control, Motivation, Go/No-Go.

## Abstract

**Rationale:**

Motivation and inhibitory control are dominantly regulated by the dopaminergic (DA) and noradrenergic (NA) systems, respectively. Hypothalamic hypocretin (orexin) neurons provide afferent inputs to DA and NA nuclei and hypocretin-1 receptors (HcrtR1) are implicated in reward and addiction. However, the role of the HcrtR1 in inhibitory control is not well understood.

**Objectives:**

To determine the effects of HcrtR1 antagonism and motivational state in inhibitory control using the go/no-go task in mice.

**Methods:**

*n* = 23 male C57Bl/6JArc mice were trained in a go/no-go task. Decision tree dendrogram analysis of training data identified more and less impulsive clusters of animals. A HcrtR1 antagonist (BI001, 12.5 mg/kg, *per os*) or vehicle were then administered 30 min before go/no-go testing, once daily for 5 days, under high (food-restricted) and low (free-feeding) motivational states in a latin-square crossover design. Compound exposure levels were assessed in a satellite group of animals.

**Results:**

HcrtR1 antagonism *increased* go accuracy and *decreased* no-go accuracy in free-feeding animals overall, whereas it *decreased* go accuracy and *increased* no-go accuracy only in more impulsive, food restricted mice. HcrtR1 antagonism also showed differential effects in premature responding, which was *increased* in response to the antagonist in free-feeding, less impulsive animals, and *decreased* in food restricted, more impulsive animals. HcrtR1 receptor occupancy by BI001 was estimated at ~ 66% during the task.

**Conclusions:**

These data indicate that hypocretin signalling plays roles in goal-directed behaviour and inhibitory control in a motivational state-dependant manner. While likely not useful in all settings, HcrtR1 antagonism may be beneficial in improving inhibitory control in impulsive subpopulations.

## Introduction

The process of decision-making involves the intricate cognitive mechanisms of information gathering and choice recognition, leading to the selection of an action from various alternatives. It encompasses several complex processes including reward evaluation (Rangel et al. 2008), motivation (Pornpattananangkul et al. 2019) and executive function (Hu et al. 2015). Impaired decision-making often manifests in patients with psychiatric and/or neurodegenerative disorders, attributed at least in part to disrupted inhibitory control, a crucial component of executive function (Gleichgerrcht et al. 2010; Cáceda et al. 2014). Clinically, this impairment can contribute to many detrimental behaviours including binge eating, drug-seeking, discontinuation of medication and poor social and financial decisions (Cáceda et al. 2014; Bossaerts and Murawski 2016). Hence, improving inhibitory control and reducing impulsivity could significantly enhance the quality of life for people with such disorders.

Two brain regions which play pivotal roles in modulating motivation and inhibitory control are the locus coeruleus (LC) and ventral tegmental area (VTA). The VTA and LC are core components of the dopaminergic (DA) and noradrenergic (NA) circuitry, respectively, with their widespread projections exerting individual and parallel influences on a diverse range of cognitive processes (Jansen et al. 2009; Ranjbar-Slamloo and Fazlali 2020). The DA system integrates reward, goal-directed behaviour, task learning and memory (Anderson et al. 2003; Wise 2004; Liu et al. 2010; Haber 2014), while the NA system is implicated in processes involving executive function and inhibitory control (Leri et al. 2002; Aston-Jones and Cohen 2005; Varazzani et al. 2015; Poe et al. 2020). Both the VTA/DA and LC/NA systems are centrally implicated in impulsivity and inhibitory control, as demonstrated in both humans and preclinical species (Loos et al. 2010; Winstanley 2011; Bari and Robbins 2013).

Hypocretin (also called orexin) neurons project widely throughout the brain, with prominent afferents to the VTA and LC. The two hypocretin peptides (Hcrt1 and Hcrt2) are exclusively produced in a relatively small population of neurons (~ 3000, ~ 7000, and ~ 50,000–80,000 in mice, rats, and humans, respectively (Modirrousta et al. 2005; de Lecea 2015)) located in the lateral and perifornical hypothalamus where they are cleaved from the common precursor protein, preprohypocretin. Hypocretins are involved in a diverse range of functions including sleep and vigilance, feeding behaviour, stress responses, learning and memory, executive function and reward (Tsujino and Sakurai 2009; Carter et al. 2010; Li and de Bin 2020; Jacobson et al. 2022). A recent cFos-based study demonstrated that greater hypocretin neuronal activation was associated with improved performance in a go/no-go task (Freeman and Aston-Jones 2020), suggesting an additional role of hypocretin neurons in inhibitory control.

Hypocretin peptides bind to two G-protein coupled receptors (HcrtR1 and HcrtR2). While HcrtR1 and HcrtR2 are expressed equally in the VTA, HcrtR1 is predominantly expressed in the LC (Li and de Bin 2020). Genetic and pharmacological studies suggest disparate roles for the two hypocretin receptors, with HcrtR2 predominantly involved in regulating sleep/wake systems (Gotter et al. 2016), whereas HcrtR1 is more involved in reward, stress and anxiety-related processes (Hopf 2020; Soya and Sakurai 2020). Moreover, a number of HcrtR1 antagonists are currently under investigation for the treatment of substance use disorders (Perrey and Zhang 2020), which are associated with impaired inhibitory control (Izquierdo and Jentsch 2012).

Previous observations have established a connection between hypocretin and feeding behaviours (Yamada et al. 2000; Bingham et al. 2006; Pankevich et al. 2010; Muthmainah et al. 2021), and between caloric restriction and motivation (Pankevich et al. 2010). Motivational state has a profound influence on the firing of DA neurons, release of dopamine, and cell signalling and gene expression in the VTA (Wilson et al. 1995; Carr 2007; Branch et al. 2013). Thus, a high motivational state, e.g., as induced by food restriction for food reward, primes the DA system to promote reward-seeking behaviour (Roitman et al. 2004). Since food restriction elevates hypocretin neuronal activity (Richardson and Aston-Jones 2012), blockade of hypocretin receptor signalling may thus decrease impulsivity induced by a high motivational state (Peleg-Raibstein and Burdakov 2021). Given the expression profile and demonstrated role of HcrtR1 in reward, motivation and addiction, this receptor may be ideally situated to mediate this effect.

The current project thus aimed to determine the effect of HcrtR1 antagonism and motivational state on impulsivity and inhibitory control in mice in a go/no-go task. This dopamine and noradrenaline-dependent operant behavioural task informs upon several aspects of executive function, including behavioural inhibition. Importantly, by manipulating caloric intake through food restriction, the effects of altered motivational state on impulsivity and inhibitory control can also be evaluated in this task. We hypothesised that HcrtR1 antagonism would increase inhibitory control in food-restricted mice, which was expected to translate in better performance in no-go trials, and decrease motivation in fed mice, resulting in lower performance in go trials.

## Materials and methods

### Drug substance

BI001 (see WO2017178339) was synthesized by Boehringer Ingelheim. A single batch of free base material was used throughout the studies. Purity was determined by NMR to be > 95%. BI001 shows reasonable physicochemical properties and aqueous solubility. In vitro, the compound shows good permeability and no P-glycoprotein-related efflux in the MDCK-MDR1 permeability assay (Madin Darby canine kidney (MDCK) cells overexpressing human Pglycoprotein), and no significant off-target stimulatory or inhibitory effects at 10µM in a standard off-target assay (Eurofins; Bowes et al. 2012). Plasma protein binding and brain tissue binding were 86.6% (fu, plasma 0.134) and 85.3% (fu, brain 0.148) as determined by Rapid Equilibrium Dialysis (RED device, Syngene International Ltd., India). Plasma protein binding was determined by equilibrium dialysis to be 87.4% (fu, 0.126). BI001 is stable in rat plasma (t1/2 > 130 min).

### Animals

All efforts were made to minimise any animal distress and/or suffering.

### Study 1: Receptor occupancy (RO%) and pharmacokinetics (PK)

Male C57BL/6NCrl mice in the weight range of 20–25 g were used for the RO% and PK studies performed at Boehringer Ingelheim GmbH (Biberach an der Riss, Germany). Mice were housed in standard wire-topped cages with *ad libitum* chow and water, wood shavings and nesting materials. The experiments were approved by the local German authorities (Regierungspräsidium Tübingen) and conducted in compliance with the German and European Animal Welfare Acts.

### Study 2: Go/No-Go task

Male C57BL/6JArc mice were sourced from the Animal Resources Centre (Perth, WA). Mice were 1.7 months of age at the start of experimentation and group-housed of up to 3 animals per cage under a 12:12 light-dark cycle, with lights off at 3pm. Mice were housed in standard wire-topped cages with *ad libitum* water, wood shavings and tissue paper nesting materials.

Experiments were performed in accordance with the Prevention of Cruelty to Animal Act (2004), the guidelines of the National Health and Medical Research Council Code of Practice for the Care and Use of Animals for Experimental Purposes in Australia (2013) and approved by the Florey Animal Ethics Committee (AEC number: 19 − 010).

### Formulation and dosing

BI001 was administered orally by gavage as a suspension. The vehicle was aqueous 0.5% Natrosol (Ashland Inc., Richmond, NSW, Australia) solution and 0.015% Tween80. Administration volume was 10 mL/kg. The BI001 suspension was prepared by either a rotor-stator homogenizer (Go/No-Go task) or a high energy focused Ultrasonicator (Covaris®, MA, US; RO% and PK).

For behavioural experiments, mice were acclimatised to oral gavage with water for one week prior to compound administration in the go/no-go task. For behavioural testing, BI001 (12.5 mg/kg) or Tween/Natrosol vehicle was administered 30 min prior to operant testing. This dose was selected based on the findings of Tyree and colleagues (2023), who showed that BI001 dose-dependently (2.5, 7.5 and 12.5 mg/kg) blocked the disinhibiting effects of optogenetic hypocretin neuron stimulation in a mouse go-no go task. For RO%, doses of 0.3, 1, 3 and 10 mg/kg were used, and for the PK study a dose of 4.4 mg/kg. All doses were administered orally.

### Blood and tissue sampling

In Study 1, trunk blood samples were collected in the RO% study directly after decapitation. For PK, serial sampling of 20 µL of blood was performed by puncture of the saphenous vein in awake animals. In both studies, blood was directly sampled into K3-EDTA coated vials. BI001 plasma and brain concentrations were also determined in a group of satellite animals to those in Study 2 after a single dose of 12.5 mg/kg of BI001, 15 or 75 min after administration (*N* = 3 per time-point). Mice were dosed as described in Sec 2.3. Mice were culled by lethal pentobarbitone overdose, at which point cardiac blood samples were collected, and brains harvested and snap frozen in isopentane. Immediately after collection of blood, plasma samples were prepared by centrifugation at 4 °C and were stored at − 20 °C (Study 1) or -80 °C (Study 2) until bioanalysis.

### Sample preparation and bioanalysis

Plasma protein was precipitated with acetonitrile. Brain samples were transferred to 7 mL Precellys® tubes and 4 parts of acetonitrile/methanol (1:1) solution were added. Samples were homogenized using a Precellys® homogenizer (Bertin technologies, France). After centrifugation, supernatants were stored at -20 °C until bioanalysis. Compound concentrations were determined by high performance liquid chromatography coupled with tandem mass spectrometry.

### In vitro pharmacology

The in vitro potency of BI001 at human HcrtR1 and HcrtR2 was assessed using an IP1 assay, as described in WO2017178339.

### Receptor occupancy

To determine receptor occupancy, mice were dosed with BI001 at 0.3, 1, 3 and 10 mg/kg or vehicle and comparisons made *versus* vehicle-treated mice. At 60 min post oral dosing, BI001-treated mice were sacrificed, and brains harvested and stored at -80 °C until processing for the assessment of receptor occupancy. Coronal brain sections containing the locus coeruleus (20 μm) were prepared using a cryostat, mounted onto glass slides and stored at -80 °C. On the day of the experiment, brain sections were thawed at room temperature and incubated in 10 nM of the HcrtR1 antagonist [^3^H]-EX5135 (Tritec, lot. nr.15-0112-0113, molecular weight, 443.5 g/mol, specific activity 55.6 Ci/mmol, WO13068935 Example 92) in a volume of 300–400 µl. Non-specific binding was defined in the presence of 10 µM cold EX5135). After a 30-min incubation at room temperature, followed by three washing steps (Tris-NaCl buffer) and a final water wash, air dried slides were incubated with film with for 4–7 days before being analysed using Aida Image Analyzer Software.

### Go/No-Go experimental procedures

#### Food intake and body weight

*N* = 24 mice were fed with standard chow *ad libitum* for seven consecutive days, at the end of which body weight was recorded as their original weight. After the seven days, food for each cage was then restricted so that the weights of mice were maintained at 90-95% of their original weight. Mice were habituated to handling for ten minutes per day prior to operant task training. Food restriction was continued during operant task training.

#### Apparatus

All operant task training and testing was conducted with 8 operant boxes (Med Associates Inc., VT, USA) in sound-attenuating chambers. Operant boxes were equipped with a grid floor, a house light (30 lx), 2 retractable levers on either side of a liquid delivery reward port, a cue light above each lever and tone generators that generated clicking sounds, variable pure tones or white noise.

#### Operant conditioning training protocol

After assessment of food intake under group-housed, basal conditions, mice entered operant conditioning training stages. 20 µL units of Strawberry milk (Iced Strawberry Milk, Nippy’s Ltd, Moorook, SA, Australia) was used as the reward throughout the study. Mice were first habituated for 3 days to the operant chambers for 30 min with strawberry milk in the reward port, where no levers or cues were presented. Mice were then trained to lever-press for a strawberry milk reward on the lever furthest from the door (the “back” lever) of the operant chamber. Mice were placed in boxes with the back lever extended during each trial for 1 h or 100 presses, whichever came first. Every time the lever was extended, a single lever press resulted in reward delivery, clicking reward sound and lever retraction for 5 s. Mice remained in this stage until they completed two sessions with > 60 responses made.

Following this, mice were trained to press the lever in response to the “go” signal used in the go/no-go task. Mice were placed in boxes for 40 min or the completion of 60 go trials, whichever came first. Every time the lever was extended, there was a “variable length non-responding phase” of 9 to 24 s, where lever presses did not trigger any response. This was followed by a 3 s “premature phase”, during which any lever press would immediately terminate the trial and lever was retracted for 10 s. After the premature phase, a 10 kHz, 75 dB pure tone go cue was played for 30 s to indicate a go trial. A lever press in the go trial phase resulted in reward delivery, clicking reward sound and lever retraction for 10 s. If no press was made during the go signal, the lever was retracted after 30 s and the 10 s intertrial interval would commence with no delivery of reward or clicker presentation. Mice remained in this training stage until they completed two sessions with greater than 45 correct go trials.

When mice met criteria with go-trial training they entered the final go/no-go task training. Mice were placed in boxes for 40 min or until completion of 60 trials (30 go trials and 30 no-go trials, presented randomly), whichever came first. Every time the lever extended, there was a “non-responding phase” and a “premature phase”, as described above for the go trial training. After the premature phase, either the pure tone identifying a go trial or a 75 dB white noise pulsed at 1 Hz that identified a no-go trial, was played. Lever presses in the go trial phase resulted in reward delivery and clicking reward sound, whereas lever presses in the no-go trial phase resulted in trial termination. The absence of level pressing during the entire 30 s no-go trial phase resulted in a reward delivery and clicking reward sound at the end of the no-go trial. Mice remained in this stage until they completed the criterion of two sessions with greater than 25 correct go trials and 10 correct no-go trials. This same task was then used for testing sessions with food restriction and HcrtR1 antagonist interventions.

#### Experimental design

**Fig. 1:**
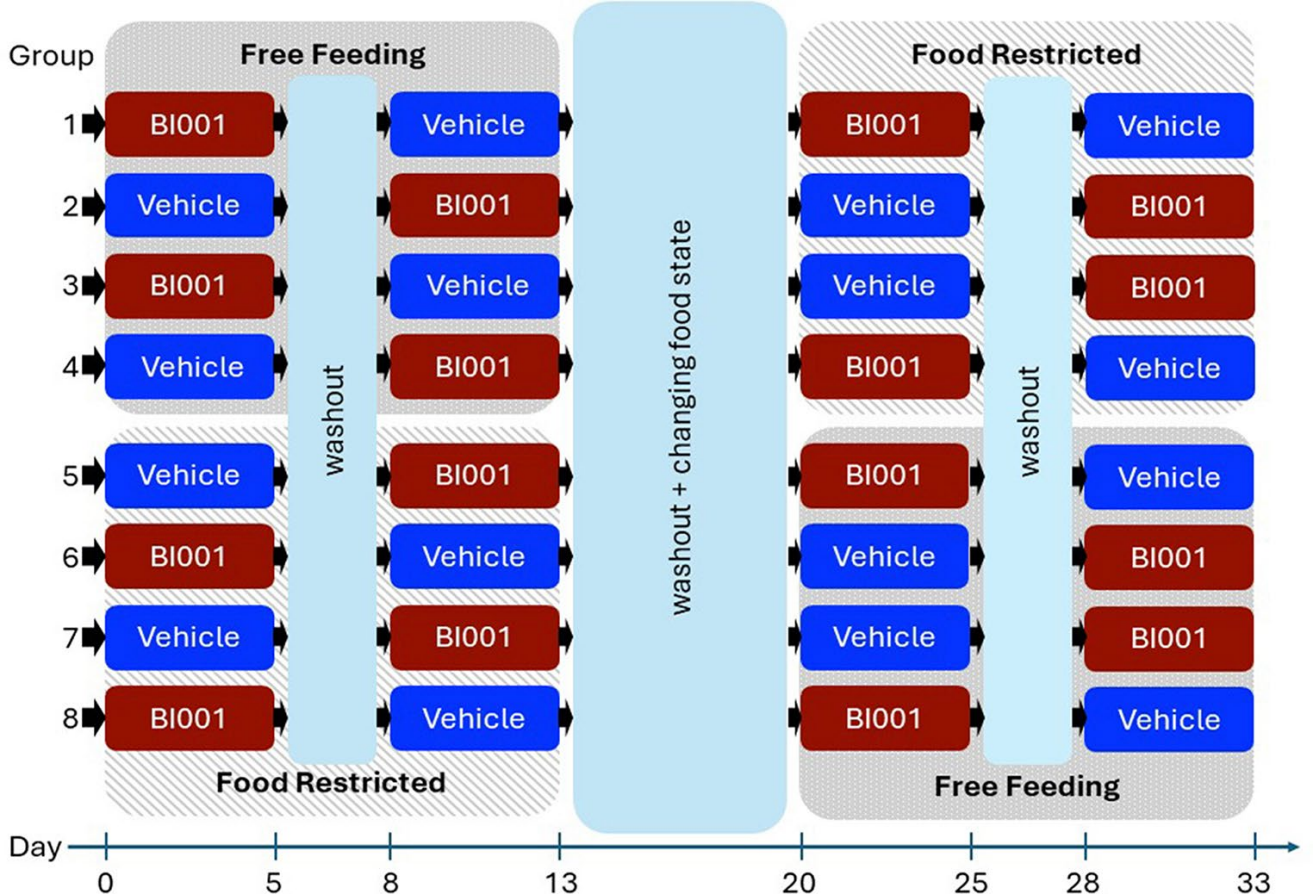
Experimental design. Mice were first allocated to 1 of 8 different treatment sequence groups. All mice then underwent all 4 treatment conditions. Free feeding (FF) and food-restricted (FR) animals underwent an initial 5-day block of drug (BI001) or vehicle (Veh) administration, followed by a 3-day drug washout period, then a 5-day block of testing with the alternate drug or Veh treatment. Next, they underwent a 7-day transition period between feeding states, followed by two additional 5-day blocks of GNG testing with BI001 or vehicle, with another 3-day drug washout period in between

A Latin square cross-over design was used with all mice undergoing all four treatment conditions: free-feeding vehicle, free-feeding HcrtR1 antagonist, food-restricted vehicle, and food-restricted HcrtR1 antagonist (Fig. 1). Mice were allocated to one of 8 groups, with each receiving a different sequence of treatments to minimise potential influences of treatment sequence. Testing in each treatment consisted of 5 consecutive days of go/no-go testing, with a three-day wash-out period between drug/vehicle transitions and seven-day transition between free-feeding/food restricted states. During washout periods, mice continued to perform daily go/no-go sessions.

To analyse performance in the go/no-go task, we measured go trial responding, no-go trial responding, non-responding presses (presses during the non-responding phase), premature presses (presses during the premature phase) and response time.

**Fig. 2:**
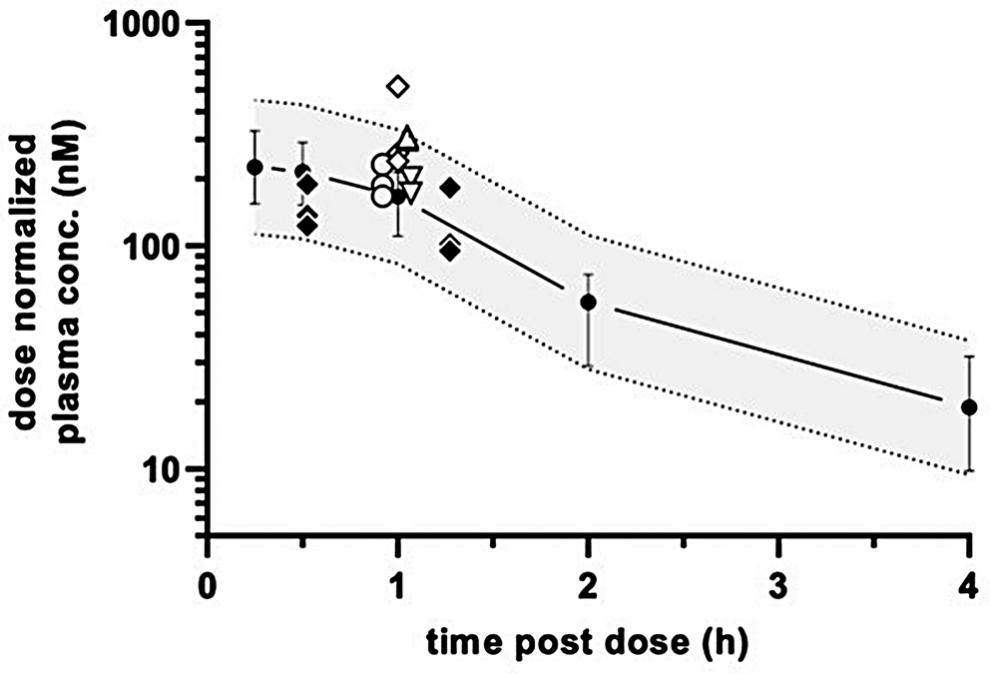
Dose-normalized plasma PK profile (closed circles, *N* = 3, ±SD) and exposure data from Study 1 RO% (open circle 0.3 mg/kg; open downward triangle 1 mg/kg; open diamond 3 mg/kg; open triangle 10 mg/kg) Study 2 (satellite) Go/NoGo animals (closed diamond). Dotted lines show 2-fold lower and higher mean PK data and illustrate that dose-normalized RO% and Go/NoGo satellite animal exposure data were, with a single exception, within a 2-fold range from the PK study mean

**Fig. 3:**
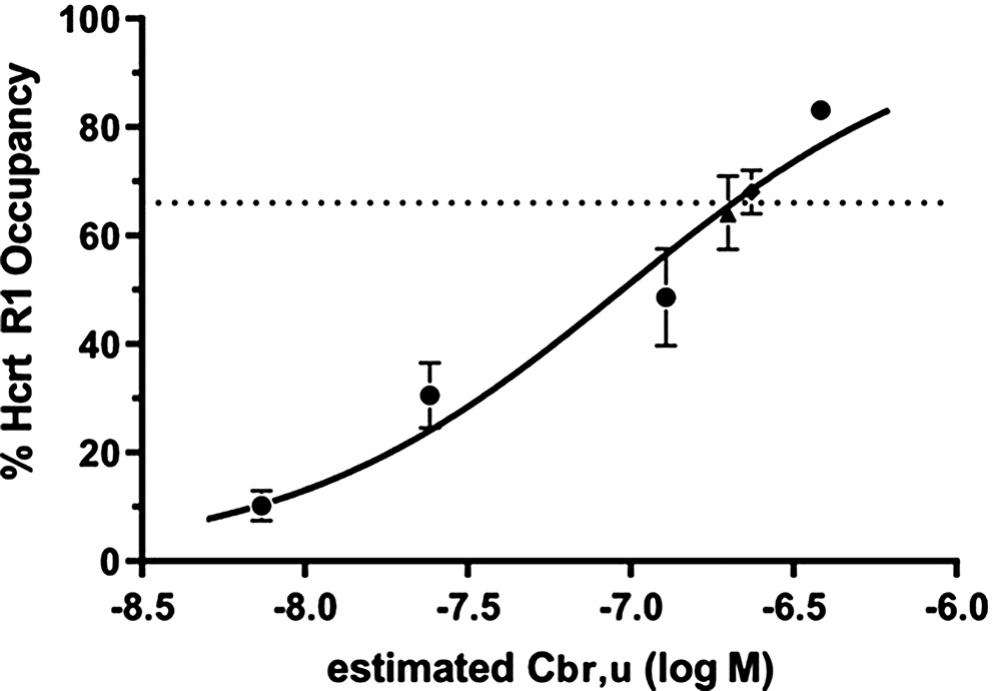
HcrtR1 receptor occupancy in the locus coeruleus measured 1 h after oral administration of BI001 at doses 0.3, 1, 3 and 10 mg/kg (solid circles. *N* = 3, ±SD). Solid line; non-linear regression with variable slope of RO data, IC_50_ 94 nM. The dotted line shows calculated Go/NoGo mean RO (66%) from 0.25 to 1.25 h post dose of 12.5 mg/kg (*N* = 3, ±SD)

### Statistical analyses

**Fig. 4:**
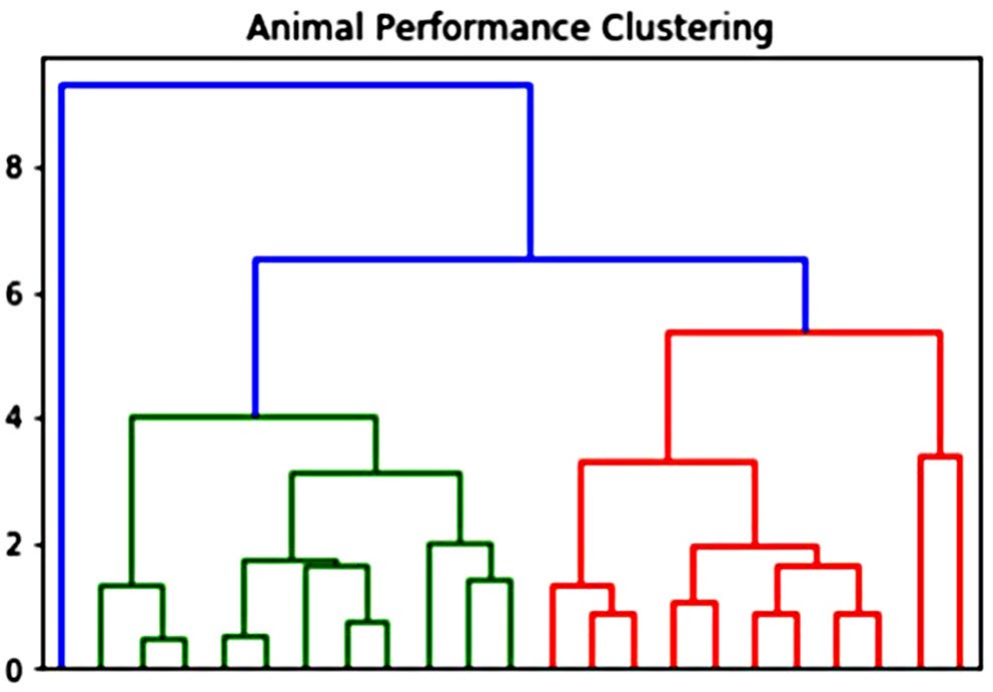
Decision tree dendrogram characterisation. The decision tree dendrogram characterised mice into two main clusters (*n* = 11 each) labelled in green and red respectively and one cluster of a single distinct mouse labelled in blue

Prior to further analyses, averaged go trial accuracy, no-go trial accuracy, premature presses, and non-responding presses from the final 10 days of no-go trial training were calculated for each mouse. Each parameter was then scaled to a standard normal distribution and a Euclidean distance matrix between animals constructed across these parameters was used to generate a hierarchical clustering dendrogram. This clustered mice into 2 major similarity clusters, one with less impulsive mice, the other with more impulsive mice (one animal was clustered by itself as distinct from either of the other clusters; Fig. 4).

Differences between cluster go accuracy, no-go accuracy, and non-responding presses were compared with unpaired t-tests. This clustering categorisation was used as a factor in subsequent statistical models to analyse go/no-go data.

Go accuracy (go correct /(go correct + go incorrect)*100), no-go accuracy (no-go correct /(no-go correct + no-go incorrect)*100), and pretrial responding ((premature + non-responding)/total *100) were analysed using logistic mixed effect regression models with feeding status (free-feeding (FF) vs. food-restricted (FR)), treatment (vehicle vs. BI001), cluster (less impulsive vs. more impulsive) and all interaction terms as fixed effects and subject random effects on the intercept.

The time taken to correctly respond by pressing a lever on a go trial (go correct response time), time to fail at withholding from pressing on a no-go trial (no-go incorrect response time) and signal detection theory parameters for sensitivity (*d’*) and response bias (*c*), calculated as d’ = *Z*(hit rate) – *Z*(false alarm rate) and. c = – 0.5 x [*Z*(hit rate) + *Z*(false alarm rate)] where *Z* is the inverse of the cumulative distribution function of the standard normal distribution, hit rate is the proportion of go trials at which a response was correctly made, and false alarm rate is the proportion of responses incorrectly made during no-go trials (McVay and Kane 2009) were analysed using linear mixed-effect regression models with feeding status (free-feeding (FF) vs. food-restricted (FR), treatment (vehicle vs. BI001), cluster (less impulsive vs. more impulsive), treatment sequence and all interaction terms as fixed effects and subject random effects on the intercept.

The most complex go/no go performance, response time, and signal detection theory parameter statistical models and all simpler sub-models were compared and the final model selection for analyses were made based on the Akaike Information Criteria (AIC), a metric balancing goodness of fit with model complexity (Portet 2020). AIC was calculated for all models and simpler sub-models, and the model with the lowest AIC selected as the best fitting model for these data. Only analyses from the final AIC-selected models are reported. Estimated marginal means were calculated for each combination of factors and post-hoc comparisons performed using z-tests with Tukey’s correction for multiple comparisons (go/no-go performance) or t-tests with Kenward-Roger degrees of freedom and Tukey’s correction for multiple comparisons (response times).

Linear and logistic regression analyses were performed using R version 4.2.2 with Rstudio (Allaire 2012) using the tidyverse, (Wickham 2016) lme4 (Bates et al. 2015), MuMIn, (Barton 2023) and emmeans (Russell et al. 2023) packages and decision tree dendrograms generated with Python 3.7.4 (Van Rossum and Drake 2012) using the scikit-learn (Pedregosa et al. 2011) and the SciPy libraries (Virtanen et al. 2020).

## Results

### In vitro pharmacology

In an IP1 assay, the Kb of BI001 at human HcrtR1 and HcrtR2 was 2.1 nM and 360 nM, respectively, indicating a selectivity for HcrtR1 over HcrtR2 of 171.

### Pharmacokinetics and pharmacodynamics of BI001

Data showed dose linearity over the dose range from 0.3 to 12.5 mg/kg (Tables 1 and 2). Observed intra- and inter-study variability was within a reasonable range (see Fig. 2). Unbound brain concentration (C_br, u_) was assumed to be equal to the unbound plasma concentration (C_pl, u_) as the compound is well permeable and shows no P-gp related efflux (see Sect. 2.1). The Go/No-Go task exposure from satellite animals in Study 2 indicated a mean RO% of 66% at the HcrtR1 over the duration of the behavioural task from 15 to 75 min post-dose (see Fig. 3). Given the 171-fold selectivity of BI001 for HcrtR1 versus HcrtR2 and the findings of Gotter and colleagues (2016), who showed a brain RO% of approximately 65% is required for efficacy at the HcrtR2, significant Hcrt2R-driven effects in the go-no go task were considered unlikely.

**Table 1:** Measured plasma concentrations and estimated unbound brain concentrations (C_br, u_) of HcrtR1 antagonist BI001. *N* = 3 animals were used for each group in Study 1 RO% and Study 2 Go/No-Go

Study	Dose (mg/kg)	Time (h)	matrix	Plasma concentration (nM)				Mean est C_br, u_ (nM)	CV%
				1	2	3	Mean		
1. RO%	0.3	1	plasma	55.8	50.1	69.4	58.4	7.36	13.9
1. RO%	1	1	plasma	202	202	172	192	24.2	7.4
1. RO%	3	1	plasma	1560	784	720	1021	129	37.4
1. RO%	10	1	plasma	2990	3090	3050	3043	383	1.4
2. Go/NoGo	12.5	0.5	plasma	2360	1710	1540	1870	236	18.9
2. Go/NoGo		1.25	plasma	1270	2280	1190	1580	199	31.4

**Table 2:** PK study (Study 1): Measured plasma concentration and estimated unbound brain concentration (est C_br, u_) after single oral administration of 4.4 mg/kg BI001 to male C57BL/6 mice. (NoP, no peak)

Time (h)	Plasma concentration (nM)				Mean est Cbr, u (nM)	CV%
	1	2	3	Mean		
0.25	1440	861	679	993	125.2	32.7
0.5	1280	669	890	946	1065.6	26.7
1	770	485	938	731	1554.1	25.6
2	127	282	328	246	768.0	35.0
4	43.1	140	65.3	83	341.6	50.1
8	2.8	26.1	2	10	52.8	108.5
24	NoP	NoP	NoP	--	--	--

**Fig. 5:**
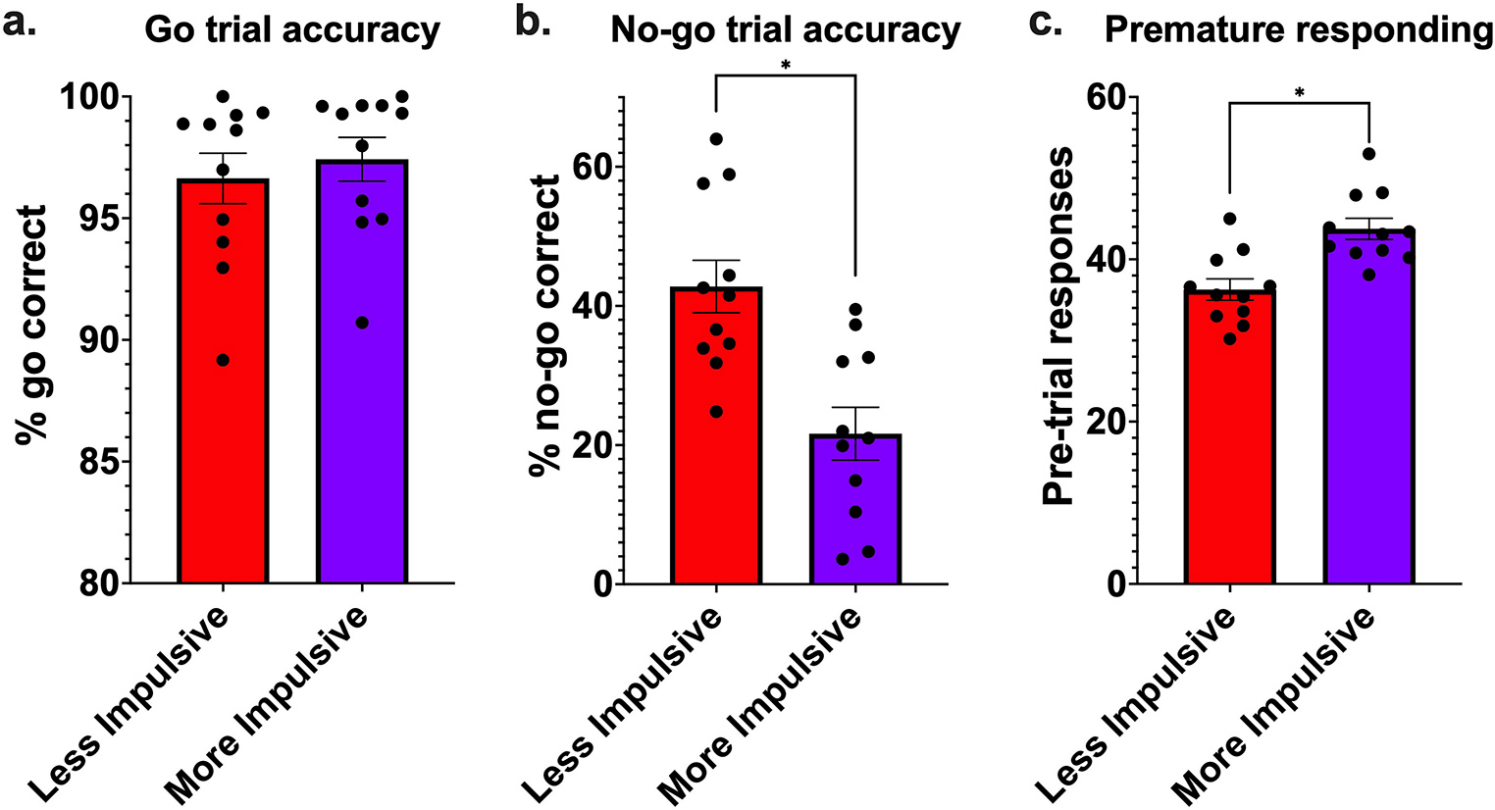
Differences between less and more impulsive animal clusters during the final training stage. (**a**) go trial accuracy; (**b**) no-go trial accuracy; (**c**) premature responding. Columns = mean ± SEM, points are individual animal data. * *p* < 0.05

### Go/No-Go task

**Fig. 6:**
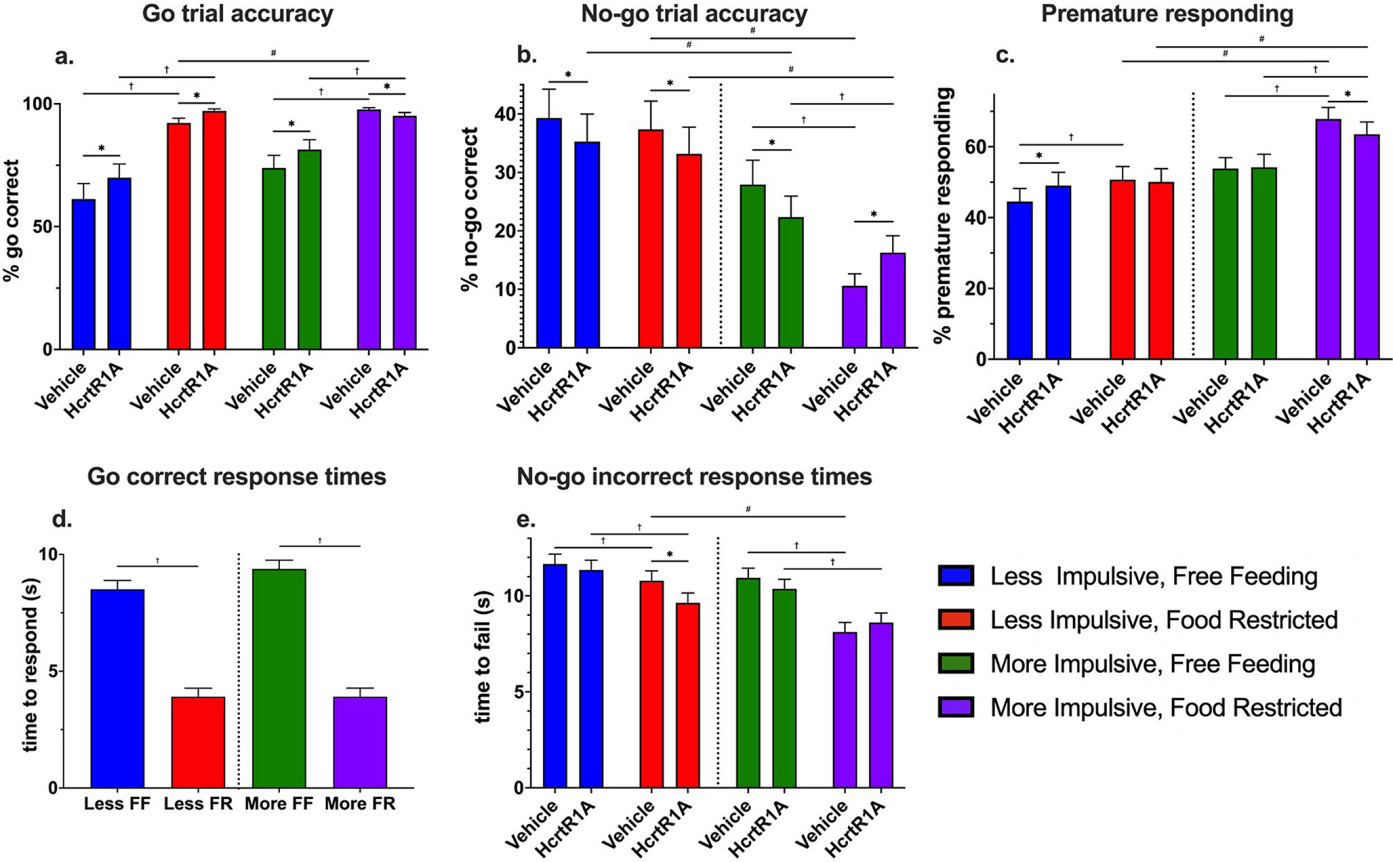
Effects of HcrtR1 antagonist BI001, motivational state, and innate impulsivity on performance of the go/no-go task. (**a**) % go accuracy (**b**) % no-go accuracy c.% premature responding. d. Successful go-trial response times (seconds). e. Failed no-go trial response times. HcrtR1A: HcrtR1 antagonist BI001. * = *p* < 0.05 HcrtR1A/vehicle differences, † = *p* < 0.05 free-feeding/food-restricted differences, # = *p* < 0.05 less/more impulsive differences. Data are model-estimated marginal means ± SEM

The results of the go/no-go task are presented as model estimated marginal means ± SEM (Fig. 6). Treatment sequence was not significant in any of the analyses, indicative that neither the efficacy of the antagonist, nor food restriction, changed across the experiment. One mouse did not complete all four treatments and was excluded from the dataset, resulting in a final sample size of *n* = 23 animals.

Mice clustered into two major similarity clusters based on training performance, with a single mouse in a third cluster branching off at an earlier point than either of the other clusters (Fig. 4). The two major clusters had two key observable differences; cluster 1 mice (*n* = 11) had a significantly lower mean no-go accuracy (22 ± 4% and 39 ± 4% for clusters 1 and 2 respectively, *p* < 0.001; Fig. 5b; Table 3), and significantly more non-responding presses (43.76 ± 4.36 and 36.27 ± 4.35 for clusters 1 and 2 respectively, *p* < 0.001; Fig. 5c; Table 3) during training compared to the cluster 2 (*n* = 11) animals There was no significant differences between the two clusters during go trials (Fig. 5a). Based on these data, cluster 1 mice were considered as more innately impulsive, and cluster 2 animals as less innately impulsive. The individual mouse clustered as more distinct from all others was excluded from subsequent behavioural analyses.

**Table 3:** Differences in pre-testing training data between decision tree dendrogram clustered groups. Data are mean ± SEM

Training differences	Cluster 1	Cluster 2	*p*
Pre-trial presses	43.76 ± 4.36	36.27 ± 4.35	< 0.05
No-go accuracy	22 ± 4%	39 ± 4%	< 0.05

#### Go accuracy

Next, we analysed go accuracy. During free feeding with vehicle dosing, there was no significant difference in go accuracy between less and more impulsive mice. Food restriction significantly increased go trial accuracy in vehicle-treated mice, whether less impulsive (61.3 ± 6.3% and 92.2 ± 2.0% for free feeding and food restricted, respectively; odds ratio = 0.1338, SE = 0.0147; z-ratio = -18.286, *p* < 0.0001; see Fig. 6a; Table 4) or more impulsive (73.9 ± 5.2% and 97.7 ± 0.7% for free feeding and food restricted, respectively; odds ratio = 0.0651, SE = 0.0121; z-ratio = -14.723; *p* < 0.0001; see Fig. 6a; Table 4), respectively.

**Table 4:** Model parameters in logistic mixed effects models during the treatment periods for go accuracy, no-go accuracy, and premature presses in the go/no-go task. The estimate “intercept” represents the predicted mean of the control group (i.e., free-feeding, vehicle-treated, less impulsive). The estimate of other effects represents the predicted change in the intercept when a specific factor or interaction is included in the model. Pr(>|t|) refers to the significance of each fixed effect using Wald tests, and AIC refers to the Akaike information criteria of the model as a whole

Response variable	Effect	Estimate	Std. Error	Pr(>|t|)	AIC
Go accuracy (%)	Go Accuracy ~ Impulsive x Food Restriction x BI001 + (1|Subject)				
	Intercept	0.459	0.264	0.0823	2860.5
	Impulsive	0.575	0.374	0.124	
	BI001	0.386	0.0852	< 0.00001	
	Food Restriction	2.011	0.110	< 0.00001	
	Impulsive x BI001	0.0549	0.125	0.660	
	Impulsive x Food Restriction	0.720	0.216	0.00083	
	BI001 x Food Restriction	0.675	0.181	0.00018	
	Impulsive x BI001 x Food Restriction	-1.901	0.293	< 0.00001	
No-go accuracy (%)	No-go Accuracy ~ Impulsive x Food Restriction x BI001 + (1|Subject)				
	Intercept	-0.434	0.206	0.0350	3500.1
	Impulsive	-0.514	0.292	0.0782	
	BI001	-0.173	0.0803	0.00311	
	Food Restriction	-0.0825	0.0806	0.306	
	Impulsive x BI001	-0.125	0.120	0.296	
	Impulsive x Food Restriction	-1.101	0.135	< 0.0001	
	BI001 x Food Restriction	-0.0120	0.115	0.916	
	Impulsive x BI001 x Food Restriction	0.804	0.186	0.00015	
Premature responses (%)	Premature Pressing ~ Impulsive x Food Restriction + Impulsive x BI001 + Food Restriction x BI001 + (1|Subject)				
	Intercept	-0.220	0.149	0.141	6602.6
	Impulsive	0.3734	0.211	0.0759	
	BI001	0.181	0.0332	< 0.0001	
	Food Restriction	0.2478	0.0333	< 0.0001	
	Impulsive x BI001	-0.167	0.0371	< 0.0001	
	Impulsive x Food Restriction	0.345	0.0374	< 0.0001	
	BI001 x Food Restriction	-0.205	0.0371	< 0.0001	

Under free feeding conditions, HcrtR1 antagonism significantly increased go accuracy in both less impulsive mice (61.3 ± 6.3% and 70.0 ± 5.6% for vehicle and HcrtR1 antagonism, respectively; odds ratio = 0.68, SE = 0.0579; z-ratio = -4.53; *p* < 0.0001; see Fig. 6a; Table 4) and more impulsive mice (73.8 ± 5.1% and 81.4 ± 4.0%; for vehicle and HcrtR1 antagonism, respectively; odds ratio = 0.643, SE = 0.586; z-ratio = -4.839; *p* < 0.0001; see Fig. 6a; Table 4). However, in the food-restricted state, HcrtR1 antagonism had contrasting effects, significantly increasing go accuracy in less impulsive mice (92.2 ± 1.9% and 97.2 ± 0.8%; for vehicle and HcrtR1 antagonism, respectively; odds ratio = 0.346, SE = 0.052; z-ratio = -6.658; *p* < 0.0001; see Fig. 6a; Table 4), but significantly decreasing go accuracy in more impulsive mice (97.7 ± 0.7% and 95.2 ± 1.3%; for vehicle and HcrtR1 antagonism, respectively; odds ratio = 2.192, SE = 0.465; z-ratio = 3.702; *p* = 0.0002 see Fig. 6a; Table 4).

#### No-Go accuracy

During free feeding with vehicle dosing, there was no significant difference in no-go trial accuracy between less impulsive and more impulsive mice. Food restriction did not significantly affect no-go accuracy in less impulsive mice, but it significantly decreased no-go accuracy in more impulsive mice (27.9 ± 4.2% and 10.6 ± 2.1% for free feeding and food restricted, respectively; odds ratio = 3.27, SE = 0.3524; z-ratio = 10.976; *p* < 0.0001; see Fig. 6b; Table 4)).

Under free feeding conditions, HcrtR1 antagonism significantly decreased no-go accuracy in both less impulsive mice (39.3 ± 4.9% and 35.3 ± 4.7% for vehicle and HcrtR1 antagonism, respectively; odds ratio = 1.19, SE = 0.954; z-ratio = 2.155; *p* = 0.0311; see Fig. 6b; Table 4)) and more impulsive mice (27.9 ± 4.2% and 22.3 ± 3.6% for vehicle and HcrtR1 antagonism, respectively; odds ratio = 1.35, SE = 0.1193; z-ratio = 3.3265; *p* = 0.0008; see Fig. 6b; Table 4)). However, in the food-restricted state, HcrtR1 antagonism had the opposite effect, significantly decreasing no-go accuracy in less impulsive mice (37.4 ± 4.8% and 33.1 ± 4.6%; for vehicle and HcrtR1 antagonism, respectively; odds ratio = 1.20, SE = 0.0985; z-ratio = 2.260; *p* = 0.0238; see Fig. 6b; Table 4)), but significantly increasing no-go accuracy in more impulsive mice (10.6 ± 2.1% and 16.3 ± 2.9%; for vehicle and HcrtR1 antagonism, respectively; odds ratio = 0.61, SE = 0.0709; z-ratio = -4.249; *p* < 0.0001; see Fig. 6b; Table 4)).

#### Premature responding

During free feeding with vehicle dosing, there was no significant difference in premature response rates between less impulsive and more impulsive mice. Food restriction significantly increased premature response rates in both less impulsive mice (44.5 ± 3.7% and 50.7 ± 3.7% for free feeding and food restricted, respectively; odds ratio = 0.781, SE = 0.026; z-ratio = -7.43; *p* < 0.0001; see Fig. 6c; Table 4) and to a greater degree in more impulsive mice (53.8 ± 3.7% and 67.9 ± 3.3% for free feeding and food restricted, respectively; odds ratio = 0.553, SE = 0.0173; z-ratio = -18.896; *p* < 0.0001; see Fig. 6c; Table 4).

Under free feeding conditions, HcrtR1 antagonism significantly increased premature response rates in less impulsive mice (44.5 ± 3.7 and 49.0 ± 3.7% for vehicle and HcrtR1 antagonism, respectively; odds ratio = 0.834, SE = 0.0277; z-ratio = -5.461; *p* < 0.0001; see Fig. 6c; Table 4) relative to vehicle treatment, but had no effect on more impulsive mice. In the food-restricted state, HcrtR1 antagonism had no significant effect on less impulsive mice, but significantly decreased premature response rates in more impulsive mice (67.9 ± 3.2 and 63.5 ± 3.5% for vehicle and HcrtR1 antagonism, respectively; odds ratio = 1.211, SE = 0.0375; z-ratio = 6.176; *p* < 0.0001; see Fig. 6c; Table 4).

#### Go reaction times

There was no significant difference in reaction times on successful go trials between less impulsive and more impulsive mice during free feeding. Food restriction significantly decreased response times in both less impulsive mice (8.49 ± 0.40 and 3.86 ± 0.37 s for free feeding and food restricted, respectively; difference estimate = 4.60, SE = 0.19; t-ratio = 24.1, *p* < 0.0001; see Fig. 6d; Table 5) and more impulsive mice (9.59 ± 0.38 and 3.81 ± 0.37 s for free feeding and food restricted, respectively; difference estimate = 5.46, SE = 0.18; t-ratio = 29.7; *p* < 0.0001; see Fig. 6d; Table 5). There were no effects of HcrtR1 antagonism in the analysed model.

**Table 5:** Model parameters in linear mixed models during the treatment periods for the reaction time of go trials with correct responses and the reaction time of no-go trials with incorrect responses. The estimate “intercept” represents the predicted mean of the control group (i.e., free-feeding, vehicle-treated and/or less impulsive, depending on the model). The estimate for other effects represents the predicted change in the intercept when a specific effect or interaction is included in the model

Response variable	Effect	Estimate	Std. Error	Pr(>|t|)	AIC
Go-trial reaction times	Go Reaction Time ~ Impulsive x Food Restriction + (1|Subject)				
	Intercept	8.510	0.362	< 0.00001	58016.7
	Impulsive	0.866	0.508	0.0998	
	Food Restriction	-4.560	0.191	< 0.00001	
	Impulsive x Food Restriciton	-0.866	0.265	0.00110	
No-go reaction times	No-go reaction times ~ Impulsive x Food Restriction x BI001 + (1|Subject)				
	Intercept	11.660	0.495	< 0.00001	53660.9
	Impulsive	-0.719	0.690	0.305	
	BI001	-0.306	0.375	0.415	
	Food Restriction	-0.867	0.386	0.0249	
	Impulsive x BI001	-0.274	0.511	0.590	
	Impulsive x Food Restriction	-1.956	0.515	0.000149	
	BI001 x Food Restriction	-0.843	0.532	0.113	
	Impulsive x BI001 x Food Restriction	1.911	0.719	0.00788	

#### No-Go reaction times

Holdout times in failed no-go trials where animals responded before the 30 s trial duration elapsed were then analysed in a similar manner. When free-feeding, there was no significant difference in time to fail on no-go trials between less impulsive and more impulsive mice. Food restriction significantly decreased holdout time in both less impulsive mice (11.66 ± 0.51 and 10.79 ± 0.51 s for free feeding and food restriction, respectively; difference estimate = 0.87, SE = 0.39, t-ratio = 2.242, *p* = 0.025; see Fig. 6e; Table 5) and more impulsive mice (10.94 ± 0.50 and 8.12 ± 0.49 s for free feeding and food restriction, respectively; difference estimate = 2.82, SE = 0.34; t-ratio = 8.268; *p* < 0.0001; see Fig. 6e; Table 5). HcrtR1 antagonism had no significant effect on time to fail in less or more impulsive mice during free feeding, but it significantly decreased time to fail in less impulsive mice during food restriction (10.8 ± 0.51 and 9.6 ± 0.51 s for vehicle and HcrtR1 antagonism, respectively; difference estimate = 1.15, SE = 0.38; t-ratio = 3.028; *p* = 0.0025; see Fig. 6e; Table 5).

#### Signal detection theory measures: sensitivity index and response bias

**Fig. 7:**
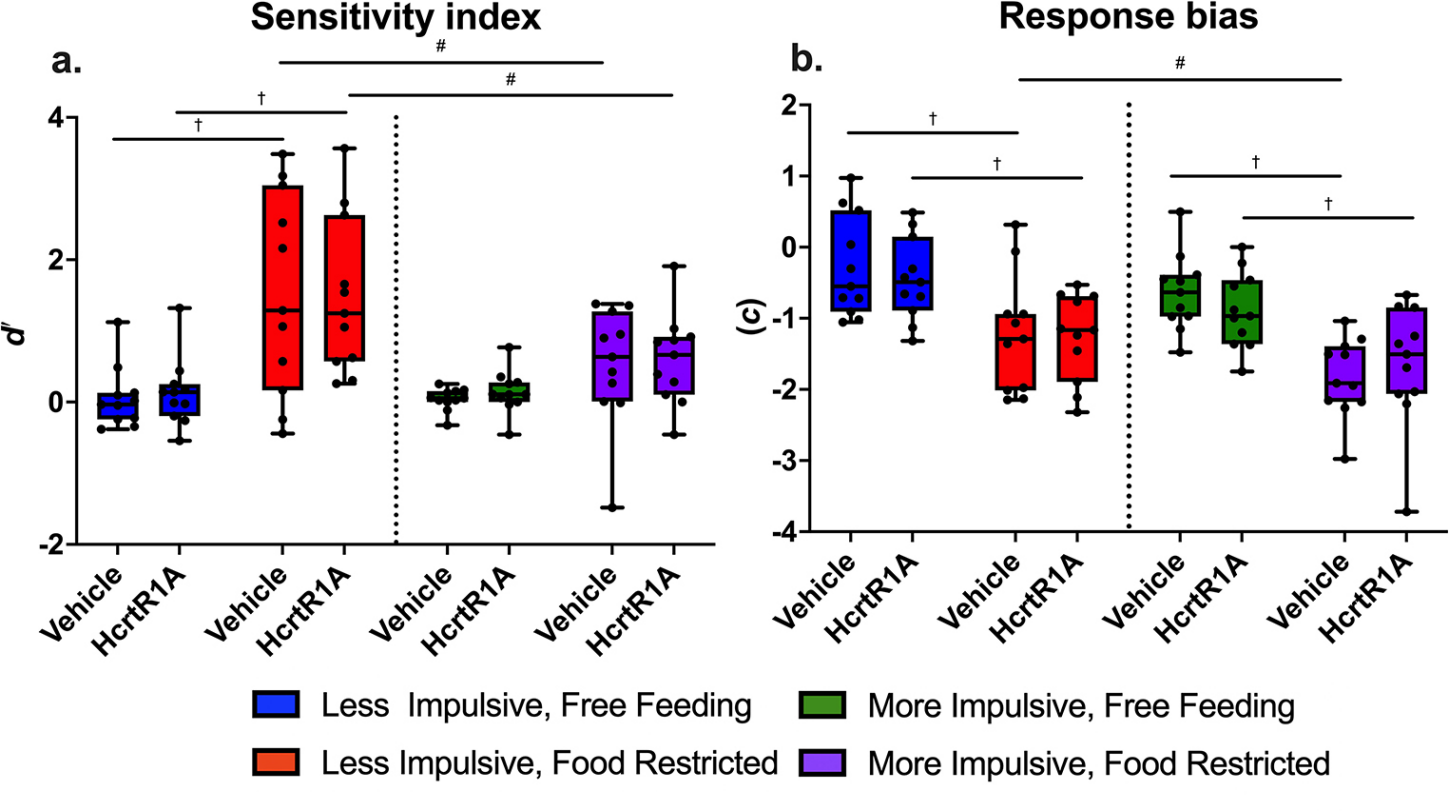
Effects of HcrtR1 antagonist BI001, motivational state and innate impulsivity on SDT sensitivity and response bias in the go/no-go task. **a**. sensitivity (***d****’*) **b**. response bias (***c***). HcrtR1A: HcrtR1 antagonist BI001. † = *p* < 0.05 free-feeding/food-restricted differences, # = *p* < 0.05 less/more impulsive differences. Boxes represent the 25th and 75th percentile with central line at median. Whiskers extend to largest and smallest values, with points representing individual animal data

 There were no significant effects of drug on sensitivity index (*d'*) in either less or more impulsive animals while free feeding or food restricted. While food restricted, more impulsive mice displayed significantly lower degrees of sensitivity than less impulsive mice under both vehicle (0.518 ± 0.235 and 1.526 ± 0.355 for more and less impulsive mice, respectively; difference estimate = 1.007, SE = 0.333; t-ratio = 3.03; *p* = 0.0032; see Fig. 7a; Table 6) and drug (0.596 ± 0.233 and 1.476 ± 0.232 for more and less impulsive mice, respectively; difference estimate = 0.881, SE = 0.321; t-ratio = 2.694; *p* = 0.0096; see Fig. 7a; Table 6) conditions. Additionally, less impulsive mice displayed significantly higher sensitivity while food restricted than when free feeding, in both vehicle (0.053 ± 0.233 and 1.526 ± 0.355 for free feeding and food restricted states, respectively; difference estimate = 1.473, SE = 0.298; t-ratio = 4.939; *p* < 0.0001; see Fig. 7a; Table 6), and drug (0.131 ± 0.350 and 1.476 ± 0.232 for free feeding and food restricted states, respectively; difference estimate = 1.346, SE = 0.284; t-ratio = 4.513; *p* < 0.0001; see Fig. 7a; Table 6), conditions.

**Table 6:** Model parameters in linear mixed models during the treatment periods for signal detection theory parameters discrimination (*d’*) and bias (*c*). The estimate “intercept” represents the predicted mean of the control group (i.e., free-feeding, less impulsive, and/or vehicle treated, depending on the model). The estimate for other effects represents the predicted change in the intercept when a specific effect or interaction is included in the model

Response variable	Effect	Estimate	Std. Error	Pr(>|t|)	AIC
Discrimination	*d’* ~ Impulsive x Food Restriction x BI001 + (1|Subject)				
	Intercept	0.053	0.235	0.821	213.4
	Impulsive	-0.013	0.332	0.968	
	BI001	0.077	0.298	0.796	
	Food Restriction	1.473	0.257	< 0.00001	
	Impulsive x BI001	0.237	0.421	0.955	
	Impulsive x Food Restriciton	-0.994	0.423	0.022	
	BI001 x Food Restriction	-0.127	0.402	0.753	
	Impulsive x BI001 x Food Restriction	0.103	0.568	0.181	
Bias	Bias ~ Impulsive x Food Restriction x BI001 + (1|Subject)				
	Intercept	-0.283	0.191	0.143	176.2
	Impulsive	-0.353	0.270	0.195	
	BI001	-0.167	0.251	0.443	
	Food Restriction	-0.953	0.216	0.00004	
	Impulsive x BI001	-0.084	0.306	0.786	
	Impulsive x Food Restriction	-0.242	0.328	0.792	
	BI001 x Food Restriction	0.133	0.313	0.666	
	Impulsive x BI001 x Food Restriction	0.299	0.433	0.492	

Similar to discrimination, there were no significant effects of BI001 administration on response bias (*c*) in either less or more impulsive animals, while free feeding or food restricted. While food restricted and vehicle treated, more impulsive mice displayed a greater liberal bias towards responding than less impulsive mice (-1.833 ± 0.220 and − 1.237 ± 0.199 for more and less impulsive mice, respectively; difference estimate = 0.596, SE = 0.283; t-ratio = 2.106; *p* = 0.0388). In both drug and vehicle treated states, food restriction also significantly increased liberal bias in more and less impulsive animals (Less impulsive, vehicle treated: -0.283 ± 0.204 and − 1.237 ± 0.199 for free feeding and food restricted mice, respectively; difference estimate = 0.954, SE = 0.268; t-ratio = 4.199; *p* = 0.0001; Less impulsive, HcrtR1A treated: -0.45 ± 0.165 and − 1.271 ± 0.219 for free feeding and food restricted mice, respectively; difference estimate = 0.821, SE = 0.227; t-ratio = 3.614; p 0.0006 L; More impulsive, vehicle treated: : -0.637 ± 0.315 and − 1.833 ± 0.220 for free feeding and food restricted mice, respectively; difference estimate = 1.196, SE = 0.217; t-ratio = 5.266; *p* < 0.0001; More impulsive, HcrtR1A treated: : -0.888 ± 0.154 and − 1.652 ± 0.200 for free feeding and food restricted mice, respectively; difference estimate = 0.764, SE = 0.326; t-ratio = 3.365; *p* = 0.0012).

## Discussion

Overall, this research shows that both food restriction motivation and HcrtR1 antagonism had significant impacts on the go/no-go performance and thus impulsivity and inhibitory behaviour in mice. Additionally, grouping animals into clusters based on their performance during go/no-go training revealed differential responses to both food restriction and HcrtR1 antagonism, depending on their innate levels of impulsivity. HcrtR1 antagonism was achieved using BI001, a highly potent and selective, brain-penetrant HcrtR1 antagonist, with oral gavage of 12.5 mg/kg BI001 providing an estimated receptor occupancy of 66% over the duration of the task. HcrtR1 antagonism significantly increased go accuracy and decreased no-go accuracy in more and less impulsive animals while free feeding, whereas it significantly decreased go accuracy and increased no-go accuracy in more impulsive, food-restricted mice. HcrtR1 antagonism also showed differential effects in premature responding, increasing in free feeding less impulsive animals, and decreasing in food-restricted more impulsive animals. Lastly, HcrtR1 antagonism decreased time to incorrectly respond in no-go trials in less impulsive, food-restricted animals.

Regarding food restriction, differences between clusters observed in the training data persisted into the testing phase in the food-restricted, vehicle-treated condition. However, differences between clusters were less apparent in the free-feeding condition. Food restriction increased go accuracy and premature responding in both less and more impulsive animals, but decreased no-go accuracy only in more impulsive animals, and increased discrimination in only less impulsive animals. Additionally, food restriction decreased the time taken to respond in both go and no-go trials, and increased liberal response bias in both less and more impulsive animals.

### Impulsivity clustering persists in testing, in a motivational state dependent manner

Different mouse strains show variation in decision-making tasks (Gubner et al. 2010; Loos et al. 2010). However, there is still innate variation in behaviour within inbred, isogenetic strains, including cognitive abilities, environment- and/or stress-related responses (Jakovcevski et al. 2008; Strekalova and Steinbusch 2010; Loos et al. 2010; Freund et al. 2013). In this study, we applied a decision tree dendrogram analysis based on training data and revealed two distinct clusters of animals in our study, with either high or low impulsivity. This clustering proved to be a robust method of separating animals as behavioural differences observed in training data, namely increased premature pressing and decreased no-go trial accuracy in the “more impulsive” persisted in the food-restricted, vehicle-treated arm of behavioural testing – the closest condition to the training environment. Additionally, once trained to meet our criteria, mice categorized into this more impulsive cluster exhibited a small but significant increase in go trial accuracy, were faster to fail on unsuccessful no-go trials, and displayed decreased response discrimination and a greater bias towards responding compared to their less impulsive counterparts, again supporting the idea that these decision tree dendrogram clusters represented less and more impulsive animals, at least in the food-restricted condition. That these differences were not observed while these animals were free-feeding and vehicle-treated suggests that the clustering was detecting a trait difference in susceptibility to food-restriction-induced increases in impulsivity, rather than a difference in trait impulsivity per se. This is similar to findings with humans that showed heightened impulsivity and inefficient response inhibition when a participant was hungry, dieting, or already showed a pre-existing preference for high calorie foods (Nederkoorn et al. 2009, 2010; Jansen et al. 2009).

### HcrtR1 antagonism has differential effects based on levels of motivation in animals

HcrtR1 antagonism showed different effects in groups of animals under different conditions, significantly increasing go accuracy and decreasing no-go accuracy in free-feeding and food-restricted less impulsive mice, and free-feeding more impulsive mice, while showing the opposite effect in more impulsive mice in the food restricted condition. If these four groups are viewed as a continuum of motivation and inhibitory control (considering food restriction to increase motivation, and cluster as a metric of innate impulsivity and inhibitory control), it is apparent that there is a differential effect of HcrtR1 antagonism between the most motivated, impulsive animals (food restricted, more impulsive cluster) and all other states. Further supporting this idea, HcrtR1 antagonism only displayed an effect on premature responding at either end of this continuum, increasing premature response rates in the free-feeding, less impulsive cluster, and decreasing premature responding in the food-restricted, more impulsive cluster. Previous studies investigating HcrtR1 antagonism in effort-based tasks (Harris et al. 2005; Borgland et al. 2009) have shown attenuated reward seeking for either palatable food or drug rewards in states of heightened motivation following both food-restriction and previous exposure to drugs of addiction. These effects are likely mediated through a HcrtR1 antagonist induced reduction in dopaminergic neuron activity.

Similarly, an observational study investigating the effects of hypocretin in the go/no-go task (Freeman and Aston-Jones 2020) showed that a higher medial hypothalamic hypocretin neuron cFos expression correlated with higher performance in go and no-go trials. However, in another hypocretin-based go/no-go study (Tyree et al. 2023), the optogenetic stimulation of hypocretin neurons resulted in elevated rates of non-responding pressing and decreased no-go accuracy. Together these findings suggest that the effects of hypocretins in the go/no-go task may follow the same inverted-U relationship observed upon treatment with DA or NA circuitry and psychostimulants (Berridge and Arnsten 2013). Highlighting the complexities of the relationship between hypocretin signalling and behavioural inhibition, another study utilising the go/no-go task in mice showed that administration of a HcrtR1 antagonist decreased non-responding presses and go accuracy, whereas it increased no-go accuracy, akin to what was observed in the highly motivated food-restricted, more impulsive group in the present study (Tyree et al. 2023), while a rat stop signal task – also performed under food restriction – showed no effect on inhibitory control indices, but did reveal a HcrtR1 inhibition driven decrease in motivation (Wiskerke et al. 2020).

Caloric restriction has previously been shown to increase the responsiveness of the hypocretin system to palatable food (Pankevich et al. 2010) and HcrtR1 and -HcrtR2 levels in the hypothalamus were increased following fasting (Mondal et al. 1999). As such, the different effects of HcrtR1 antagonism across groups could be explained by these interactions; this suggests that while not globally effective at improving inhibitory control, HcrtR1 antagonism may be beneficial in subpopulations where hypocretin levels are elevated beyond what is optimal, including individuals with obesity and binge eating disorder or addictions, who often exhibit high trait impulsivity (Guerrieri et al. 2008; Piccoli et al., 2012; Mitchell and Potenza 2014; Bénard et al. 2017; Giel et al. 2017; Rømer Thomsen et al. 2018; Gómez-Martínez et al. 2022), in conjunction with elevated motivational state during episodes of craving (Sinha 2013; Verzijl et al. 2022). Indeed, these observations of differential efficacy of HcrtR1 antagonism parallel findings within the addiction landscape, where an interesting body of work utilising a behavioural economics approach to study drug seeking has shown that HcrtR1 antagonism is most efficacious at attenuating cue induced reinstatement behaviours for both cocaine (James et al. 2019b; a) and opioids (Fragale et al. 2019; Mohammadkhani et al. 2019) in animals with elevated demand elasticity, i.e., those that were more driven to expend effort for these rewarding substances. Furthermore, increased lateral hypothalamic hypocretin cell number and activity following cocaine exposure was observed in those animals with elevated demand elasticity and sensitivity to HcrtR1 antagonism (James et al. 2019b). This supports the hypothesis that elevated hypocretin signalling in the more impulsive animals could have contributed to their increased impulsivity while food restricted, thus reducing this signalling back to normal levels through HcrtR1 antagonism restored performance on no-go trials.

### Food restriction increases motivation, and decreases response inhibition in more impulsive mice

When food-restricted, all mice exhibited greatly elevated reward motivation as indicated by significantly increased go accuracy and decreased response times on go-correct trials. This is likely due to an increase in the perceived value of the reward (strawberry milk) in the food restricted state. Previous studies have shown increased motivation to work for reward in food-restricted states (Dixon et al. 2014), and that food-related stimuli are perceived as more pleasant and evoke stronger insula and inferior occipital responses in a fasted than fed state (Uher et al. 2006). Also related, food restriction appeared to decrease response inhibition in this task, based on no-go accuracy and premature responding rates and response times, as might be expected in conjunction with the heightened reward drive in the food-restricted state (He et al. 2014), but to different degrees depending on the innate impulsivity, with larger inhibitory control deficits evident in the innately more impulsive animals. Similar observations have also been made in human studies, with trait impulsivity and food craving interactively impacting food-related behavioural inhibition (Diergaarde et al. 2009; Meule and Kübler 2014; Velázquez-Sánchez et al. 2014). Additionally, food restriction increased liberal bias towards responding in both less and more impulsive animals. This increase in response bias again supports the idea of food restriction decreasing inhibitory control and driving animals towards reward seeking behaviours, potentially through making other reward related cues (for example, the strawberry milk aroma in the operant chamber, or presentation of reward linked lever) more salient, as has been observed in obesity (Loeber et al. 2012) and thirst (Freeman et al. 2014). Food restriction also increased sensitivity albeit only in the less impulsive subset of animals. That this is only observed in the less impulsive animals is likely due to the relatively larger decrease in no-go accuracy observed in the more impulsive group. Together, these data suggest that inhibitory control in this task relies on the interplay between top-down executive function and bottom-up reward motivation, and additionally that innately more impulsive individuals may be more susceptible to inhibitory control deficits induced by altered motivational states.

Interestingly, when compared to food-restricted C57BL6/J mice from other go/no-go studies (Gubner et al. 2010; Loos et al. 2010; Jones et al. 2017; Freeman and Aston-Jones 2020; Tyree et al. 2023), these mice showed comparatively poorer performance on no-go trials. This is likely due to the difference in length of no-go trials between the present paradigm (30 s) and the aforementioned studies (5–10 s), presenting a substantially harder task during no-go trials. However, given that mean times to fail in no-go trials across all groups were around 8–12 s in length, it is likely that shorter no-go trials would not have provided the dynamic range required to observe drug- or food restriction-induced differences in no-go accuracy.

## Conclusion

In conclusion, this research shows that in a subpopulation of highly motivated, highly impulsive individuals, HcrtR1 antagonism may be a viable strategy for rescuing and restoring inhibitory control, while proving ineffective at elevating inhibitory control above baseline in a more general population.

Additionally, this research highlights the interplay between top-down executive function and bottom-up reward motivation (as altered by caloric restriction) in modulating inhibitory control; our study suggests that individuals with higher trait impulsivity may be more susceptible to inhibitory control deficits induced by a heightened motivational state.
